# Gut Microbiota Contribute to Heterosis for Growth Trait and Muscle Nutrient Composition in Hybrid Largemouth Bass (*Micropterus salmoides*)

**DOI:** 10.3390/microorganisms13071449

**Published:** 2025-06-22

**Authors:** Jixiang Hua, Qingchun Wang, Yifan Tao, Hui Sun, Siqi Lu, Yan Zhuge, Wenhua Chen, Kai Liu, Jie He, Jun Qiang

**Affiliations:** 1 Wuxi Fisheries College, Nanjing Agricultural University, Wuxi 214081, China; huajixiang@foxmail.com (J.H.); taoyifan@ffrc.cn (Y.T.); sunhhh0126@126.com (H.S.); liuk@ffrc.cn (K.L.); 2021113003@stu.njau.edu.cn (J.H.); 2 Suzhou Aquatic Technology Extension Station, Suzhou 215004, China; chun22315@163.com (Q.W.); yanzhuge2024@163.com (Y.Z.); chenwenhua215@163.com (W.C.); 3 Key Laboratory of Freshwater Fisheries and Germplasm Resources Utilization, Ministry of Agriculture and Rural Affairs, Freshwater Fisheries Research Center, Chinese Academy of Fishery Sciences, Wuxi 214081, China; lusiqi@ffrc.cn

**Keywords:** hybridization, microbiota taxa, amino acid, fatty acid, non-additive

## Abstract

Crossbreeding often results in heterosis. In this study, we generated hybrids from largemouth bass from geographically isolated populations. Growth, digestive enzyme activity, and muscle nutrient composition were compared between the hybrid groups (NC and CN) and the purebred groups (NN and CC), and the gut microbiota was investigated. The heterosis rates of body length, body height, and body thickness in hybrid largemouth bass were statistically significant. Digestive enzyme activity was higher in the hybrid groups than in the purebred groups. Compared with the CN and CC groups, the NC group had significantly higher levels of essential amino acids and total amino acids in the muscle. The polyunsaturated fatty acid content was lower in the hybrid groups than in the purebred groups. The gut microbiota in the hybrid groups predominantly exhibited a non-additive inheritance pattern, characterized by a reduced abundance of Proteobacteria and an increased abundance of Fusobacteria. Microbial taxa showing differences in abundance between the hybrid and purebred groups harbored genes enriched in multiple amino acid and fatty acid metabolism pathways. *Cetobacterium*, *Pseudomonas*, and *Stenotrophomonas* were more abundant in the hybrids, and were positively correlated with multiple amino acids and fatty acids. These results highlight the critical role of gut microbiota in heterosis.

## 1. Introduction

Heterosis refers to the phenomenon where the F_1_ hybrid generation derived from parents with distinct genetic backgrounds exhibits enhanced growth rates or superior stress resistance compared with its parental lines [1]. Hybridization has emerged as a routine and effective strategy in the breeding of various livestock [2,3], crops [4,5,6], and aquatic animals [7,8,9], significantly enhancing breeding efficiency and production performance. An understanding of the mechanism of heterosis is critical for determining its potential benefits in agricultural technology and biotechnology. Extensive research has focused on the molecular mechanism of heterosis from a genetic perspective [10,11,12]. Nevertheless, it is likely that many other factors also contribute to heterosis.

The complex array of microbes in the gut, collectively termed the gut microbiota, maintains a long-term, intimate, and intricate relationship with the host [13]. The gut microbiota represents a highly specialized and structurally complex microbial ecosystem, which is shaped by interactions among microorganisms, the host, dietary intake, and environmental factors. Host phylogeny and dietary patterns are recognized as two predominant factors influencing the composition and structure of the gut microbial community in animals [14,15]. The gut microbiota plays a crucial role in the development of the host’s nervous system, behavioral regulation, immune function, food digestion, and metabolic processes [16]. It also influences host reproduction by affecting gametic integrity and embryo viability, potentially playing a critical role in the formation of new species [17,18]. Previous research has shown that the gut microbiota can impact the host genome, thereby altering the host’s physiological and metabolic activities. This enables hosts to expand their dietary niches and access novel nutritional opportunities, and suggests that gut microbiota may contribute to the process of speciation [17]. For example, Acetitomaculum was found to be significantly increased in the crossbred progeny of sika deer (*Cervus nippon*) and elk (*Cervus elaphus*), enhancing their nutrient absorption and utilization [19]. Huang et al. [20] proposed that alterations in the intestinal microbial structure of crossbred progeny represent a key factor contributing to heterosis. Consequently, the gut microbiota is one of the potential physiological mechanisms underlying heterosis.

The gut microbiota plays a crucial role in facilitating the digestion and absorption of nutrients, thereby influencing the nutrient composition of muscle. Muscle nutrient composition is a critical parameter for evaluating the nutritional value of aquatic products [21]. The lipid content in muscle significantly influences its elasticity and texture [22]. The presence of certain amino acids and fatty acids not only determines whether the muscle can supply essential nutrients required by humans, but also plays a crucial role in determining muscle flavor [23,24], providing significant advantages in terms of quality and marketability. Breeding to improve the nutritional profile of muscle holds significant potential. Several studies have shown that the crossbred progeny exhibit marked differences from their parental lines in terms of lipid, amino acid, and fatty acid contents in muscle [25,26], suggesting that hybridization is an effective method for breeding progeny with improved muscle nutritional profiles. These findings imply that there are critical relationships among the gut microbiota, genome, and phenotype. The genome of hybrid progeny may allow them to develop distinct gut microbiota, which contributes to the heterosis of certain phenotypic traits and muscle nutrient composition.

Largemouth bass (*Micropterus salmoides*) is an economically important fish species. Its advantages include rapid growth, strong stress resistance, and flavorful and nutritious meat [27]. Nevertheless, reduced genetic diversity and differentiation in genetic structure have been widely observed in the breeding populations of largemouth bass [28]. Although several cross-breeding studies have aimed to improve the genetics of largemouth bass [29,30], they did not fully elucidate the mechanisms of heterosis in this fish. Although research into the crossbreeding of aquatic animals has been ongoing since the last century, the theoretical foundation underlying heterosis remains enigmatic [31,32,33]. In this study, largemouth bass from geographically isolated populations were utilized as parental lines. The phenotypic traits, muscle nutrient composition, and gut microbial structure of the crossbred progeny were systematically analyzed and compared. The aims of this study were to elucidate the mechanistic basis of heterosis in largemouth bass from the perspective of gut microbiota, and to investigate the influence of hybridization on the muscle nutrient profile. The results of this study provide a theoretical foundation for the genetic improvement of largemouth bass in both research and practical settings.

## 2. Materials and Methods

### 2.1. Experimental Populations

Two geographically isolated populations of largemouth bass were utilized as parental lines for cross-breeding: the US-introduced protospecies (Wild population in the freshwater basin of Florida) and the farmed population from Jiangsu, China. Two crossbred progeny populations were generated through hybridization. Specifically, one crossbred progeny group (NC) was produced with the US-introduced protospecies as the paternal parent and the farmed population from Jiangsu as the maternal parent. The other crossbred progeny group (CN) was generated with the farmed population from Jiangsu as the paternal parent and the US-introduced protospecies as the maternal parent. The progeny groups resulting from the interbreeding of two parental groups were utilized as the controls. The purebred group derived from the US-introduced protospecies was designated as NN, and the purebred group derived from the farmed population was designated as CC.

In accordance with the principle of pairing one female fish with two male fish, groups of three fish were placed into the breeding facility for spawning based on the hybridization design described above. Each experimental group was established using 20 distinct parental pairs to ensure population genetic diversity and enhance the reliability of the experimental results. After the fish fry were reared to a length of 10 cm, 180 individuals with an average weight of 4.82 ± 0.02 g were selected for the breeding experiment in each group. Twelve cages (2 m × 2 m × 2 m) were installed in the breeding pond, with 60 individuals placed in each cage. Each group consisted of three replicate cages. During the experiment, the dissolved oxygen concentration in the water remained above 6 mg/L, the total ammonia nitrogen was 0.50 ± 0.12 mg/L, and the pH was 7.67 ± 0.26. Fish were fed a complete diet of commercial feed (with a crude protein content of ≥48% and a crude fat content of ≥6%) twice daily (at 8:00 a.m. and 5:00 p.m.). The experiment lasted for 180 days.

### 2.2. Phenotypic Measurement and Sample Collection

At the end of the 180-day experimental period, the fish were fasted for 24 h, and then 30 fish per replicate (90 fish per group) were randomly selected for analysis. Growth traits, including body length, body height, and body thickness, were measured. Heterosis, expressed as a percentage (*H*%), was calculated for each phenotypic trait as described by Mai et al. [10], with the following formula:

$$H\%=\frac{{\bar{F}}_{1}-\left(\bar{P_{M}}+\bar{P_{F}}\right)/2}{\left(\bar{P_{M}}+\bar{P_{F}}\right)/2}\times 100\%$$

where *F*_1_, *P_M_*, and *P_F_* represent the average phenotypic values of the hybrid population, paternal line, and maternal line, respectively. The significance of the *H*% value was tested using the one-way ANOVA followed by the Tukey test, with a significance level set at *p* < 0.05. After the morphological indices were recorded, 12 fish were randomly selected from each group. The fish were anesthetized using MS-222, and muscle tissue samples (four pieces per parallel) were collected from the dorsal muscle at 2 cm posterior to the head. The muscle samples were stored at −20 °C until nutrient composition analysis. In addition, 15 fish were randomly selected for analyses of digestive enzyme activity and the gut microbiome. The intestinal tissue was promptly dissected, and its surface was moistened with 8.5‰ *w*/*v* normal saline solution. Then, the contents of the posterior half of the intestine were collected, immediately frozen in liquid nitrogen, and stored at −80 °C until the gut microbial analysis. After removing the intestinal contents, the cleaned intestinal tissue was frozen in liquid nitrogen and stored at −80 °C until digestive enzyme activity assays.

### 2.3. Determination of Digestive Enzyme Activity

The intestinal tissue was homogenized with nine volumes of normal saline to prepare a 10% *w*/*v* tissue homogenate. The homogenate was centrifuged at 2500 rpm for 15 min at 4 °C using a high-speed centrifuge (Model 17/17R, Thermo Fisher, Waltham, MA, USA). The supernatant was collected, and the activities of amylase, lipase, and proteinase were measured using commercial assay kits (Jiancheng Institute, Nanjing, China) according to the manufacturer’s instructions.

### 2.4. Proximate Composition of Muscle Samples

Muscle nutrient composition was analyzed as described previously [22]. The muscle moisture content was determined by the drying method, specifically by calculating the difference in weight between before and after drying under 101.3 kPa air pressure at 105 °C. The muscle crude protein content (with a 6.25 N-to-protein conversion factor) was measured using the Kjeldahl method. The crude fat content was assessed by Soxhlet extraction. The muscle ash content was determined after incineration at 550 °C using a furnace (Carbolite Gero, Sheffield, UK).

### 2.5. Determination of Muscle Amino Acid and Fatty Acid Composition

The amino acid concentration in muscle was determined using an automatic amino acid analyzer (Model LA8080, Hitachi, Tokyo, Japan). Lyophilized muscle samples were hydrolyzed with 6 mol/L hydrochloric acid at 110 °C. After two cycles of drying and redissolution, each sample was dissolved in 1 mL of diluent and the pH was adjusted to 2.2 with sodium citrate buffer. The resulting solution was then filtered through a 0.22 μm membrane prior to analysis. Amino acid concentrations were quantified by calculating the peak areas using the external standard method.

The lyophilized muscle sample was hydrolyzed using a hydrochloric acid solution, then neutralized with an ethanol solution, and extracted with an ether-petroleum ether mixture. The extracts were then concentrated and dried using a rotary evaporator (Model N-1300S, EYELA, Tokyo Rikakikai Co., Ltd., Tokyo, Japan) to obtain total muscle lipids. Fatty acid methyl esters (FAMEs) were prepared by reacting the mixture with 2% *w*/*v* sodium hydroxide in formaldehyde, using triglyceride nondecarbonate as the internal standard. Qualitative analysis of FAMEs was performed using a gas chromatograph (Model 7890A, Agilent, Santa Clara, CA, USA) equipped with a capillary column (length: 100 m, inner diameter: 0.25 mm, film thickness: 0.2 μm). The gas chromatography procedure followed the method described by Wang et al. [22]. The fatty acid content was quantified using the peak area percentage method. The significance testing of muscle nutrition was analyzed using SPSS 26.0 (SPSS Inc., Chicago, IL, USA). Homogeneity of variance and normality were performed for the data. One-way analysis of variance (ANOVA) was conducted for the data meeting the criteria. Significant differences were assessed using Duncan’s multiple comparison test, with *p* < 0.05 considered statistically significant.

### 2.6. Nucleic Acid Extraction and High-Throughput Sequencing

Gut microbes were analyzed as described by Wang et al. [34]. Genomic DNA was extracted from intestinal microbial samples using the EZNA Stool DNA Kit (D4015, Omega Inc., Norcross, GA, USA). The DNA extracted from three fish from the same group were mixed to prepare the sequencing library (five replicates for each group). Following confirmation of DNA quality, the extracted DNA was amplified using universal primers for the v3-v4 region: 341F (5′-ACTCCTACGGGAGGCAGCA-3′) and 805R (5′-GGACTACHVGGGTWTCTAAT-3′). After amplification, the PCR products were purified using AMPure XP beads (Beckman Coulter Genomics, Danvers, MA, USA) and then quantified using a Qubit fluorometer (Invitrogen, Carlsbad, CA, USA). The sequencing libraries were prepared using the Pacific Biosciences SMRTbell^TM^ Template Prep Kit (Kapa Biosciences, Woburn, MA, USA). Library quality was assessed using an Agilent 2100 Bioanalyzer (Agilent), and sequencing was performed on the Illumina NovaSeq platform (Illumina, San Diego, CA, USA).

### 2.7. Data Preprocessing

After sequencing, the raw reads were quality-filtered using fqtrim (v0.94) to obtain high-quality clean reads. The sequences were denoised following the QIIME2 DADA2 workflow to eliminate invalid mosaic sequences and extract amplicon sequence variants (ASVs). Subsequently, clustering was performed using Vsearch software v2.3.4., and the feature sequences of each ASV were classified using the classify-sklearn algorithm in QIIME2. The α-diversity of microbiota was calculated based on the ASV abundance table using QIIME2. The Kruskal–Wallis rank sum test and Dunn’s test were used to assess the significance of differences among groups. Additionally, QIIME2 was used to compute the β-diversity of microbiota, and parametric and non-parametric statistical tests were performed using R packages. Multi-level taxonomic classification of the gut microbiota was conducted by referencing the NT-16S database and utilizing SILVA138 (https://www.arb-silva.de/documentation/release-138/; accessed on 6 October 2024), with the significance evaluated accordingly.

### 2.8. Inheritance Patterns of Microbial Abundance

A Kruskal–Wallis H test was conducted on all gut microbiota detected at the gene level to evaluate differences in their relative abundance between the hybrid and purebred groups. The microflora showing significant differences in abundance between these groups were selected for further analysis of their inheritance patterns. Following the methods of Huang et al. [20] and Mai et al. [10], the microbial taxa were categorized into three patterns of inheritance: additive, non-additive (dominant), or nonadditive (over-dominant). Based on the differences in microbial abundance between the hybrid and purebred groups, the microbiota was categorized into 12 paternal-cross-maternal abundance patterns. Specifically, when there was a significant difference in microbial abundance between the parental groups, and the abundance in hybrid groups was between that in the two parents, this inheritance pattern was referred to as additive (Patterns I and XII). When the microbial abundance in the hybrid population did not significantly differ from that of one parent population but was significantly higher or lower than that in the other parent population, its inheritance pattern was classified as dominant (Patterns II, IV, IX, and XI). When the microbial abundance in the hybrid groups was significantly higher or lower than that of both parental populations, its inheritance pattern was defined as over-dominant (Patterns V, VI, VIII, III, VII, and X).

### 2.9. Correlation Analysis

Key bacterial genera that exhibited a significantly increased abundance in the hybrid group were identified. The Mantel test was employed to conduct correlation analyses between microbial taxa and the contents of muscle amino acids and fatty acids, as well as digestive enzyme activities. The correlation analysis between specific amino acids or fatty acids was performed using the Spearman rank correlation test. A significant correlation was defined as occurring when *p* < 0.05.

## 3. Results

### 3.1. Enhancement of Growth Performance

**Figure 1:**
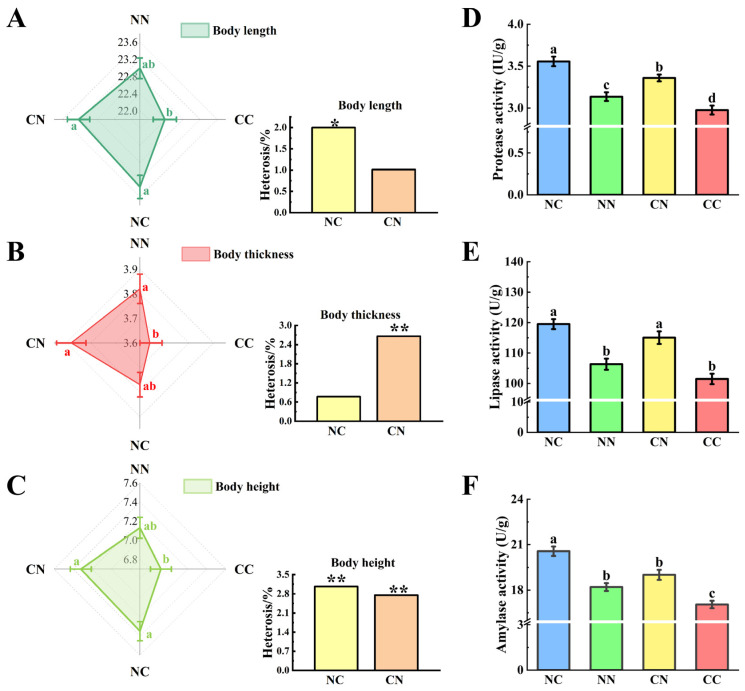
Comparative analysis of growth and digestive enzyme activities between hybrid and purebred groups. Assessment of differences and heterosis (%) in terms of body length (**A**), body thickness (**B**) and body height (**C**). Different small letters indicate statistically significant differences among four groups. Values marked with * and ** indicate statistically significant significance at *p* < 0.05 and *p* < 0.01. Enzyme activity of (**D**) protease, (**E**) lipase and (**F**) amylase. NC and CN, hybrid groups; CC and NN, purebred groups.

The phenotypic characteristics of the hybrid groups were significantly different from those of the purebred groups (Figure 1A–C). Specifically, the values of body length, body thickness, and body height were markedly higher in the hybrid groups (NC and CN) than in the CC group (*p* < 0.05). The values of these phenotypes were also higher in the NC and CN than in the NN group; however, the differences were not statistically significant (*p* > 0.05). The calculation of heterosis rates for each phenotype revealed significant heterosis of body length and body height in the NC group (*p* < 0.05); and body thickness and body height in the CN group (*p* < 0.05). Notably, body height exhibited the highest heterosis rate (3%).

We analyzed the digestive enzyme activities in gut, including protease activity (Figure 1D), lipase activity (Figure 1E), and amylase activity (Figure 1F). The enzymatic activities of the hybrid group were significantly different from those of the purebred group (*p* < 0.05). Notably, the activities of all three digestive enzymes were significantly higher in the NC group than in the two purebred groups (*p* < 0.05); and the activities of protease and lipase were significantly higher in the CN group than in the two purebred groups (*p* < 0.05).

### 3.2. Differences in Proximate Composition of Muscle

Proximate analyses of muscle samples revealed that (Table 1) the water and protein contents in the muscle were similar among the four groups, with no significant differences (*p* > 0.05). The fat content in the muscle was higher in the hybrid groups than in the purebred groups (*p* < 0.05). The ash content was significantly higher in the NC group than in the CN group (*p* < 0.05).

### 3.3. Differences in Amino Acid and Fatty Acid Composition of Muscle

The amino acid composition of the muscle of largemouth bass in the four populations is shown in Table 2. With the exception of Gly, all the other amino acids showed significant differences in their contents in muscle among the four groups (*p* < 0.05). The amino acid profiles of the NC group and NN group were similar, and all amino acids were present at higher levels in these groups than in the CC group (*p* < 0.05). The levels of Ser, Glu, Tyr, Arg, Pro, Cys, and Met did not differ significantly between the CN group and the NC or NN groups (*p* > 0.05). However, the levels of other amino acids were significantly lower in the CN group than in the NC and NN groups (*p* < 0.05). In addition, the levels of essential amino acids, delicious amino acids (DAA), and total amino acids were significantly higher in the NC and NN groups than in the CN and CC groups (*p* < 0.05). Notably, the CC group exhibited the lowest levels of amino acids, which were significantly lower than their corresponding levels in the CN group (*p* < 0.05). The ratio of essential amino acids to total amino acids was comparable across all groups, with no significant differences (*p* > 0.05).

The fatty acid composition of the muscle in largemouth bass for each group is shown in Table 3. A total of 15 fatty acids were identified in this study. The saturated fatty acid content was significantly higher (*p* < 0.05) in the NC group than in the other groups. The monounsaturated fatty acid content was highest in the CN group, significantly higher than that in the NC, NN, and CC groups (*p* < 0.05). Additionally, the polyunsaturated fatty acid content in the muscle was significantly lower in the hybrid groups than in the purebred groups (*p* < 0.05). The fatty acid composition profile of the NC group was similar to that of the NN group, but was similar between the CN group and the CC group. Compared with the other groups, the NC group demonstrated significantly lower levels of n-6 polyunsaturated fatty acids (*p* < 0.05), and the CN group exhibited significantly reduced levels of n-3 polyunsaturated fatty acids (*p* < 0.05).

### 3.4. Differences in Gut Microbial Community Composition

**Figure 2:**
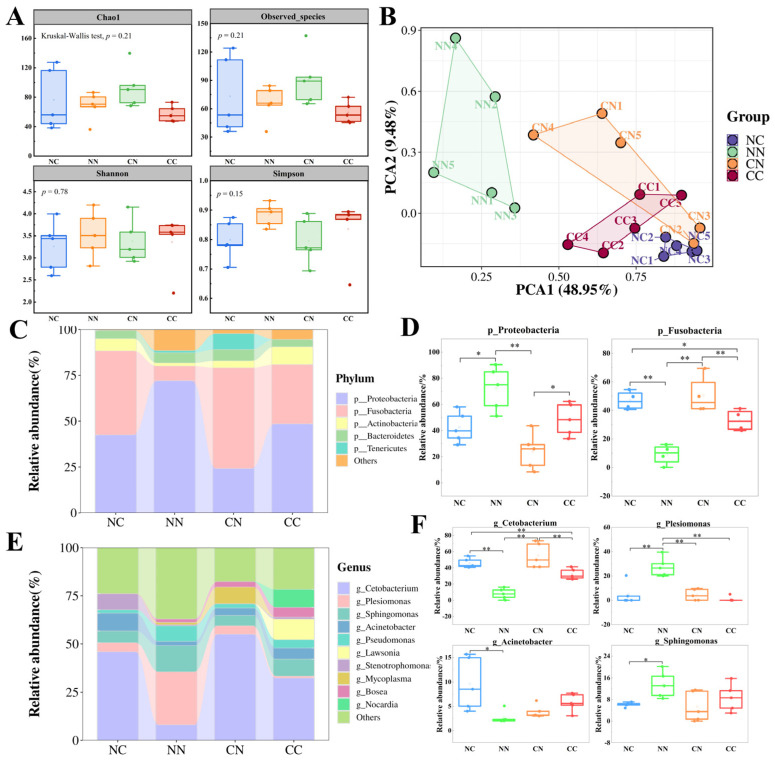
Comparison of gut microbial community composition between hybrid groups and purebred groups. (**A**) Analysis of gut microbial α-diversity in hybrid and purebred largemouth bass. Measures of α-diversity included Chao1 index, observed species index, Shannon index and Simpson index. (**B**) Principal component analysis based on weighted_unifrac distance calculation. (**C**) Overall composition of microbial community at phylum level; top 5 taxa in terms of relative abundance are shown. (**D**) Relative abundance of dominant phylum-level bacteria. * and ** indicate statistically significant difference at *p* < 0.05 and *p* < 0.01. (**E**) Overall composition of microbial communities at genus level. (**F**) Relative abundance of dominant bacterial genera.

The α-diversity of the intestinal microbial communities was compared on the basis of the Observed_species index and Chao1 index, which are indicators of the species richness of gut microbiota (Figure 2A). The values of these indexes were similar in all the groups, with no significant differences (*p* > 0.05). Shannon’s index and Simpson’s index, which are indexes of biodiversity, were higher in the CC and NN groups than in the NC and CN groups, but the differences were not statistically significant (*p* > 0.05). We conducted a principal coordinates analysis based on weighted UniFrac distances to evaluate the β-diversity of intestinal microbiota. As shown in Figure 2B, the samples from the four groups formed distinct clusters. Specifically, the NN group formed a cluster that was separate from the other groups, whereas the clusters for the NC, CN, and CC groups were located close together, with partial overlap between the CC and CN groups. These findings suggest that the structure of the intestinal microbial community differed among the four groups. Consequently, we further investigated the composition of gut microbiota in the four groups.

First, the structure of the intestinal microbiome in the hybrid groups and purebred groups was analyzed at the phylum level (Figure 2C, Table S1). The results showed that Proteobacteria and Fusobacteria were the most important microbial taxa in the largemouth bass intestine. The abundance of these two phyla differed significantly between the hybrid groups and the purebred groups (*p* < 0.05, Figure 2D). Specifically, Proteobacteria exhibited the highest relative abundance in the NN group (72%), which was significantly higher than that in the other groups (*p* < 0.05). The relative abundance of Proteobacteria was lowest in the CN group (24.16%). The relative abundance of Fusobacteria was significantly higher in the NC group (45.87%) and CN group (54.99%) than in the NN group (8.05%) and CC group (32.35%) (*p* < 0.05). Additionally, the NC and CC groups exhibited a higher abundance of Actinobacteria, while the CN group exhibited a higher abundance of Tenericutes.

Next, the composition of gut microbiota was analyzed at the genus level (Table S2). *Cetobacterium* was the predominant genus in the largemouth bass intestine (Figure 2E); its relative abundance was significantly higher (*p* < 0.05) in the NC and CN groups (45.87% and 54.98%, respectively) than in the NN and CC groups (8.04% and 32.35%, respectively). The highest relative abundance of *Plesiomonas* was in the NN group, at 27.44%, which was significantly higher (*p* < 0.05) than its abundance in the two hybrid groups and the CC group. Additionally, *Stenotrophomonas* accounted for 8.3% of gut bacteria in the NC population, whereas its abundance was nearly negligible in the other groups. Except for *Cetobacterium* and *Plesiomonas*, among the four genera with the highest abundance (Figure 2F), *Acinetobacter* exhibited the highest abundance in the NC group, significantly higher than in the NN group (*p* < 0.05). *Sphingomonas* showed the highest abundance in the NN group, significantly higher than in the NC group (*p* < 0.05). However, the abundance of *Sphingomonas* did not differ significantly between the CN group and the CC group (*p* > 0.05).

### 3.5. Inheritance Patterns of the Microbiome in Hybrid Groups

**Figure 3:**
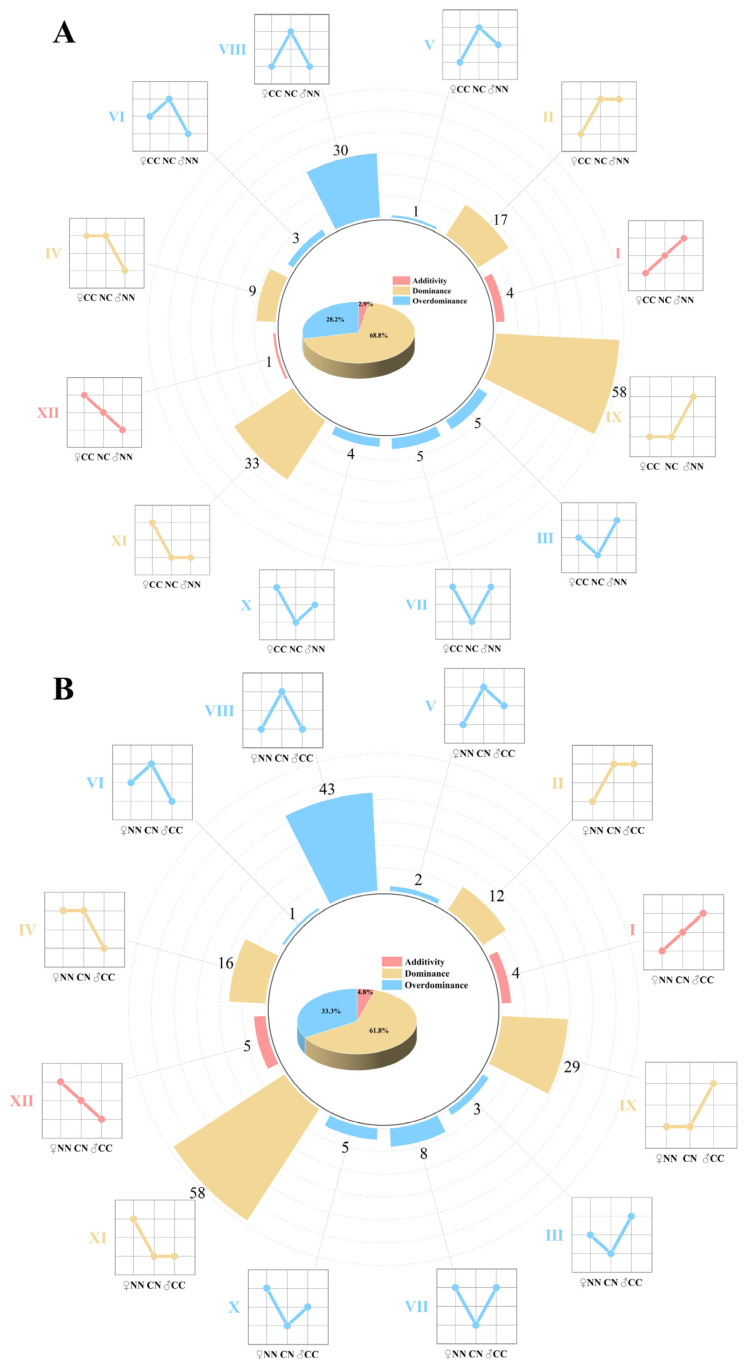
Inheritance patterns of gut microbiome. (**A**) Inheritance patterns between NC group and parental lines. (**B**) Inheritance patterns between CN group and purebred group. Microbiome was divided into 12 inheritance patterns based on differences in abundance; additive (I, XII), dominant (II, IV, IX and XI), and over-dominant (III, V, VI, VII, VIII and X). Line charts illustrate differences in abundance between hybrid and purebred groups.

The relative abundance of intestinal microbes in four groups of largemouth bass was analyzed at the genus level. A total of 228 genera with significant differences in abundance among the groups were identified. Based on the variations in abundance among the different groups, 170 bacterial genera in the NC group and 186 bacterial genera in the CN group were categorized into 12 distinct abundance patterns. These 12 patterns were further classified into three inheritance types: additive (I and XII), dominant (II, IV, IX, and XI), and over-dominant (III, V, VI, VII, VIII, and X). In the NC group (Figure 3A), five, 117, and 48 microbial taxa were categorized into additive, dominant, and over-dominant modules, respectively, and these accounted for 2.9%, 68.8%, and 28.2% of the total bacterial community, respectively. In the CN group (Figure 3B), nine, 115, and 62 microbial taxa were classified into additive, dominant, and over-dominant modules, representing 4.8%, 61.8%, and 33.3% of the total count, respectively. Notably, the dominant modules were most prevalent in the hybrid groups.

### 3.6. Functional Analysis of Microbiome

**Figure 4:**
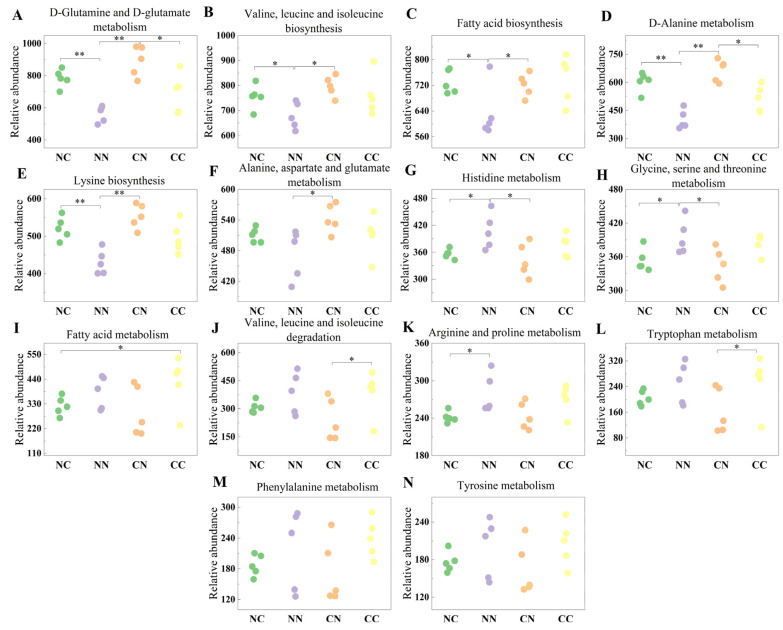
Functional analysis of gut microbial genes showing significant differences in abundance in between hybrid and purebred groups. Relative abundance of microbial genes involved in (**A**) D-Glutamine and D-glutamate metabolism; (**B**) Valine, leucine and isoleucine biosynthesis; (**C**) Fatty acid biosynthesis; (**D**) D-Alanine metabolism; (**E**) Lysine biosynthesis; (**F**) Alanine, aspartate and glutamate metabolism; (**G**) Histidine metabolism; (**H**) Glycine, serine and threonine metabolism; (**I**) Fatty acid metabolism; (**J**) Valine, leucine and isoleucine degradation; (**K**) Arginine and proline metabolism; (**L**) Tryptophan metabolism; (**M**) Phenylalanine metabolism; (**N**) Tyrosine metabolism. Values marked with * and ** indicate statistical significance of *p* < 0.05 and *p* < 0.01.

To further investigate the roles of gut microbiota, we employed PICRUSt2 software v2.6.0. to derive relative gene abundance information from the DNA sequence data. The obtained sequence information was compared against the KEGG database (Table S3). The results showed that the gut microbiota was associated with numerous pathways regulating amino acid and fatty acid metabolism (Figure 4). Specifically, compared with the purebred groups, the hybrid groups showed an increased abundance of microbial genes involved in D-Glutamine and D-glutamate metabolism; valine, leucine, and isoleucine biosynthesis; fatty acid biosynthesis; D-Alanine metabolism; and lysine biosynthesis (*p* < 0.05). The relative abundance of microbial genes involved in histidine metabolism and glycine, serine, and threonine metabolism was significantly higher in the NN group than in the hybrid groups (*p* < 0.05). In addition, the hybrid and purebred groups exhibited differences in the relative abundance of microbial genes involved in other pathways associated with amino acid metabolism, although these differences were not statistically significant (*p* > 0.05).

### 3.7. Correlations Between Gut Microbiome and Muscle Nutrient Composition

**Figure 5:**
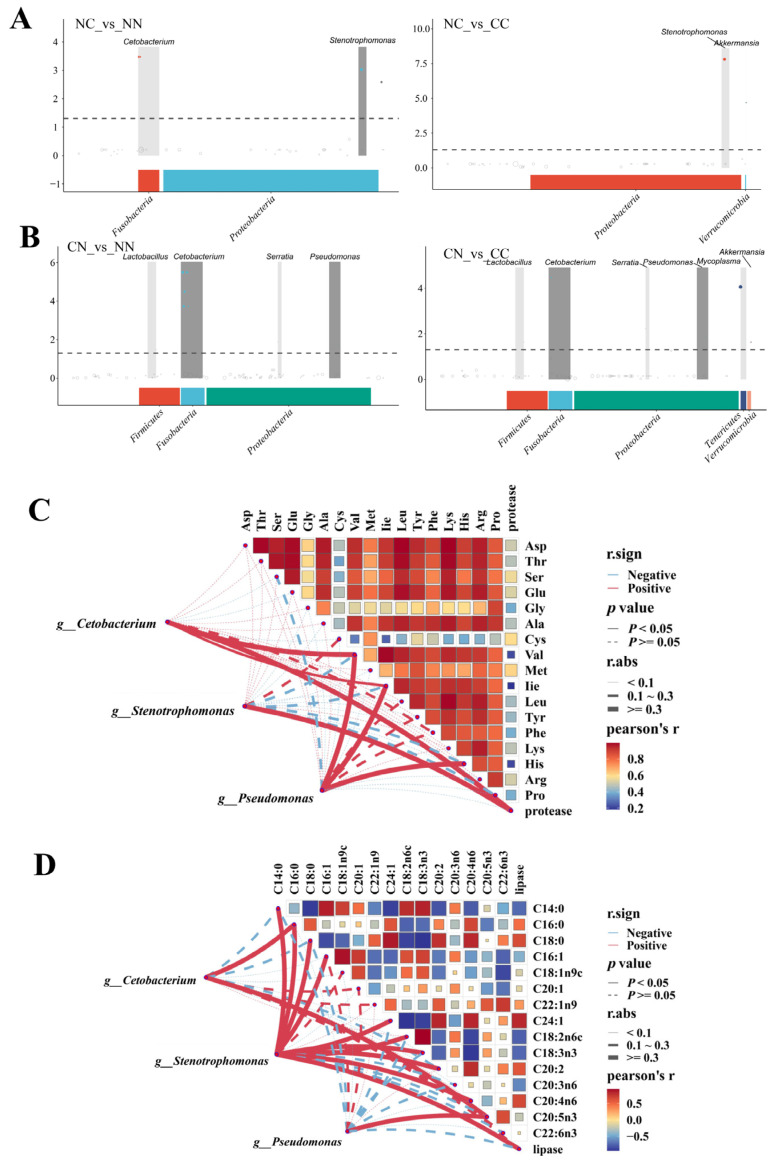
Associations between microbiome and muscle nutrients. (**A**) Manhattan map based on MetagenomeSeq analysis between NC group and parental lines. (**B**) Manhattan map based on MetagenomeSeq analysis between CN group and parental lines. Horizontal axis represents annotation of ASV at phylum level; vertical axis indicates −log10(adj-Pvalue) value. The dashed line denotes threshold for significance level of the difference. Correlation network heatmap represents relationships between genera and (**C**) amino acids and (**D**) fatty acids. Correlation analysis of network was conducted using Mantel’s test; correlation analysis of the heat map was conducted by Spearman’s test. Red and blue indicate positive and negative correlations, respectively.

Based on the MetagenomeSeq analysis, we identified key microbiota taxa exhibiting significantly increased abundance in the hybrid groups. As shown in Figure 5A, the abundance of *Cetobacterium* was significantly higher in the NC group than in the purebred groups, and the abundance of *Stenotrophomonas* was significantly higher in the NC group than in the NN group (*p* < 0.05). Both *Cetobacterium* and *Pseudomonas* were significantly more abundant (*p* < 0.01) in the CN group than in the purebred group (Figure 5B) and *Lactobacillus* and *Serratia* were significantly more abundant in the CN group than in the purebred group (*p* < 0.05). The majority of microbiota taxa showing significantly higher abundance in the hybrid groups than in the purebred groups belonged to the Proteobacteria and Fusobacteria phyla.

*Cetobacterium*, *Pseudomonas*, and *Stenotrophomonas* were selected to investigate the correlations between microbial genera and the contents of amino acids and fatty acids and the activity of digestive enzymes. For these three microbial taxa, their abundance was significantly associated with the amino acid content in the muscle of largemouth bass (Figure 5C). Specifically, the abundance of *Cetobacterium* was significantly correlated with Val content, while the abundance of *Pseudomonas* was significantly correlated with the contents of Val, Ile, and His (*p* < 0.05). Both *Cetobacterium* and *Stenotrophomonas* were significantly correlated with protease activity (*p* < 0.05). The abundance of *Stenotrophomonas* exhibited significant correlations with multiple fatty acids (*p* < 0.05, Figure 5D), including C14:0, C16:0, C18:0, C16:1, C24:1, C18:2n6c, C18:3n3, C20:2, C14:3, C14:2, and C20:4n6, as well as lipase activity. Both *Cetobacterium* and *Pseudomonas* were significantly associated with C16:0 and C20:5n3 (*p* < 0.05). All of the detected correlations were positive. In addition, in pairwise comparisons, most amino acids were strongly positively correlated with each other, whereas fatty acids showed relatively weak correlations.

## 4. Discussion

Hybridization allows for the combination of alleles from parents with different genotypes, thereby enhancing the genetic diversity of the crossbred progeny, and allowing them to inherit superior traits from both parents or exhibit traits superior to those of both parents [11]. The evaluation of the heterosis rate of target traits and analyses of the underlying genetic mechanisms have biological significance and are also relevant for production. Heterosis has been exploited to enhance economically important traits, such as growth performance and nutritional quality. The heterosis rate serves as a valuable metric for assessing phenotypic heterosis [10,35]. In this study, the *H*% values of the NC group and CN group reached statistically significant levels in terms of body length, body thickness, and body height. These findings suggest that the hybrid fish in this study exhibited positive heterosis.

The muscle nutrient profile is an important indicator of fish product quality [36]. Previous studies have demonstrated that the muscle nutrient composition of aquacultured animals can be improved through hybridization [37,38]. In this study, the lipid content of muscle was significantly higher in the hybrid groups than in the purebred groups. Based on the results of our previous study, we propose that the increased muscle lipid content in the hybrid groups may enhance the flavor of the meat [22]. The amino acid profiles of muscle were similar between the NC group and NN group, but these two groups showed significantly higher total amino acid and essential amino acid contents compared with the CN group and the CC group. Compared with the parental lines, the hybrid groups exhibited significantly lower polyunsaturated fatty acid contents in muscle. Additionally, the level of monounsaturated fatty acids in muscle was significantly lower in the NC group than in the other groups. Therefore, the NC group in this study inherited the superior amino acid composition from the paternal line and exhibited a more favorable amino acid profile compared with that of the maternal line. However, in the CN group, the amino acid and fatty acid contents in muscle generally demonstrated negative heterosis, a phenomenon that has been reported in other studies [39,40]. It is important to understand the mechanisms of positive heterosis, but equally important to understand those of negative heterosis. Considering the critical role of gut microbiota in nutrient digestion and absorption, we expected that gut microbial functions may differ among the various groups, and these differences may help to explain the heterosis of muscle nutrient profiles.

Because the gut microbiota is influenced by factors such as diet and environment, its composition and structure exhibit significant variations among populations. The host’s genetic information is one of the critical factors influencing the structure of the gut microbial community [14,15]. Previous studies have demonstrated that hybrid aquatic species, including hybrid abalone (*Haliotis laevigata* × *Haliotis rubra*) [41], hybrid Chinook salmon (*Oncorhynchus tshawytscha*) [42], and whitefish (*Coregonus clupeaformis*) [43], display markedly different gut microbial communities compared with those of their parental lines. In this study, there was no significant difference in the α-diversity of the gut microbial community among the four groups, suggesting that the gut microbiota of largemouth bass is more stable than those of other aquatic animals. In another study, the gut microbes of fish subjected to stress conditions displayed relatively stable richness and evenness [34], a finding that aligns with this study. The β diversity is a key indicator of the composition and structure of gut microbiota [44]. Based on the consistency of the water environment maintained in all groups, we conclude that despite the uniformity in microbial abundance observed across all groups, the composition of the gut microbial community differed between the hybrid groups and the purebred groups. Regarding the influence of the water environment on the composition and structure of the gut microbiota in largemouth bass, we will develop another research protocol to conduct a comprehensive and systematic analysis. Notably, digestive enzyme activity was significantly higher in the hybrid groups than in the purebred groups. Digestive enzymes play critical roles in the absorption and utilization of nutrients [45]. We found that certain microbial taxa exhibited significant positive correlations with digestive enzyme activity, suggesting that changes in the abundance of particular gut microbes in the hybrid groups may underlie the increased digestive enzyme activity and the changes in muscle nutrient composition.

Non-additive inheritance plays a crucial role in the formation of heterosis [46,47]. To determine its contribution to heterosis of largemouth bass, we categorized the bacterial genera showing significant differences in abundance between the hybrid and purebred groups into 12 genetic inheritance patterns, in three classes: additive, non-additive (dominant), and nonadditive (over-dominant). The majority of microbial taxa in the two hybrid groups showed non-additive inheritance patterns, consistent with the findings of Mai et al. [10] and Huang et al. [20]. The non-additive inheritance of gut microbiota in largemouth bass may be associated with the development of heterosis. Notably, the proportion of bacteria showing non-additive inheritance was lower in the NC group (2.9%) than in the CN group (4.8%). Accordingly, compared with the CN group, the NC group showed marginally superior growth performance and muscle nutrient composition. This shows that non-additive inheritance of gut microbiota may significantly affect the growth and muscle nutrient composition of hybrid largemouth bass [48,49]. Functional annotation analyses showed that a large number of microbial taxa potentially contribute to the synthesis and metabolism of amino acids and fatty acids, with significant differences observed in their relative abundance across groups. Thus, the differences in microbial abundance in the hybrid groups compared with the purebred groups may serve as a critical factor contributing to the differences in muscle nutrient composition between these groups.

Proteobacteria and Fusobacteria are significant components of the gut microbiota in bony fish [50,51]. In this study, these two phyla were identified as the dominant microbial groups among the four groups of largemouth bass, with their combined relative abundance exceeding 70%. Tan et al. [52] found that Proteobacteria was the predominant microbial group in largemouth bass. Similarly, Jin et al. [53] found that the relative abundance of Proteobacteria reached over 90% in the microbiota of largemouth bass. These findings underscore the absolute dominance of Proteobacteria and Fusobacteria in the gut microbiota of largemouth bass. Proteobacteria include numerous genera or strains that are pathogenic or opportunistic pathogens, which are generally considered as unfavorable for fish survival [54,55]. Proteobacteria are thought to negatively affect their hosts because members of this phylum contain a large number of genes encoding virulence factors and antibiotic resistance factors [56]. However, this conclusion warrants further scrutiny. The physiological roles and functions of Proteobacteria in fish have not yet been comprehensively investigated. Other studies have demonstrated a direct proportional relationship between the abundance of Proteobacteria and the amino acid and fatty acid contents in fish [57]; and a relationship between the high abundance of Proteobacteria in short-intestinal fish and enhanced metabolism of amino acids and lipids [58]. These findings suggest that Proteobacteria may play a role in metabolic processes related to energy and material metabolism in fish. Fusobacteria have been demonstrated to exhibit a positive correlation with the levels of lipids, amino acids, and other nutrients in fish [59], and may potentially contribute to the bioaccumulation of toxic substances [52]. In this study, the Proteobacteria and Fusobacteria in the gut microbiota of largemouth bass were likely involved in the biological processes associated with amino acid and fatty acid synthesis and metabolism. Notably, previous studies have shown that the abundance of Proteobacteria and Fusobacteria is generally negatively correlated [52,54,60]. Li et al. [61] proposed that Proteobacteria and Fusobacteria/Firmicutes/Bacteroides can be categorized into two distinct functional groups, which exhibit functional differences in carbohydrate utilization, short-chain fatty acid production, virulence factors, and antibiotic resistance. Furthermore, Li et al. [61] suggested that under non-stress conditions, the ratio of functional group 2 (Fusobacteria, Firmicutes, Bacteroides) to functional group 1 (Proteobacteria) is positively correlated with fish growth. These findings suggest that there are interactions between Proteobacteria and Fusobacteria. In this study, hybrid populations with a higher relative abundance of Fusobacteria exhibited similar characteristics.

We also conducted an in-depth analysis of the differences in microbiome composition at the genus level between the hybrid and purebred groups. We detected significantly higher abundance of *Cetobacterium*, *Stenotrophomonas*, *Pseudomonas*, *Lactobacillus*, and *Serratia* in the hybrid groups than in the purebred groups. These microbial taxa may be associated with heterosis. Previous studies have demonstrated that *Cetobacterium* is able to synthesize essential amino acids, including Leu, Ile, Val, and Gly. Furthermore, it can utilize carbohydrates for polysaccharide biosynthesis and improve carbohydrate utilization in fish [62,63]. *Stenotrophomonas*, which was significantly enriched in the NC group, may have promoted the growth and development of fish in this group. Although some studies have suggested that *Stenotrophomonas* may be a pathogen associated with certain fish diseases [64,65], there is probably a more complex relationship at play. In fact, a positive correlation was detected between the abundance of *Stenotrophomonas* and the growth rate of fish [66]. Mondal et al. [67] demonstrated that the abundance of *Stenotrophomonas* increased significantly in infected rohu (*Labeo rohita*). Furthermore, *Stenotrophomonas* quickly became a dominant genus in the gut of sheepshead minnow (*Cyprinodon variegatus*) during exposure to environmental stress [68]. These findings indicate that *Stenotrophomonas* may enhance the host’s growth and/or play a role in its stress response. Similarly to *Stenotrophomonas*, *Pseudomonas* is commonly recognized as a pathogen affecting aquacultured animals [69]. However, *Pseudomonas* has also been shown to exhibit probiotic properties [70].

The mechanisms by which particular microbes influence the growth and metabolism of fish warrant further investigation. In this study, *Cetobacterium*, *Stenotrophomonas*, and *Pseudomonas* exhibited positive correlations with the contents of multiple amino acids and fatty acids. Notably, the abundance of *Stenotrophomonas* was significantly positively correlated with the levels of various fatty acids. The relationship between the gut microbiota and muscle nutrient profiles has been well established in previous studies [71]. Our results indicate that these microbial taxa, particularly *Stenotrophomonas*, may play crucial roles in determining the composition of amino acids and fatty acids in the muscle of largemouth bass. Interestingly, these three microbial taxa belong to Proteobacteria and Fusobacteria, emphasizing the important role of these phyla in promoting the growth of largemouth bass and in determining the amino acid and fatty acid profiles of its muscle tissue. However, the precise mechanisms by which the aforementioned microbial taxa influence heterosis remain unclear. In our subsequent research, we will use the findings of this study to investigate the roles of Proteobacteria and Firmicutes in heterosis, exploring potential pathways for addressing heterosis-related challenges via gut microbiota.

## 5. Conclusions

Crossbred progeny derived from two geographically isolated parental lines exhibited significant heterosis in several phenotypic traits, including body length, body thickness, and body height. Compared with the purebred groups, the crossbred progeny demonstrated enhanced digestive enzyme activity, altered muscle nutrient composition, and differences in the abundance of gut microbes, primarily following a non-additive pattern of inheritance. These changes were significantly different from those in the purebred groups, and were significantly correlated with variations in phenotype and muscle nutrient profiles. The relative abundance of microorganisms harboring genes enriched in KEGG pathways associated with amino acid and fatty acid synthesis and metabolism exhibited significant differences between the hybrid and purebred groups, which contributed to the differences in muscle nutrient profiles between these groups. Proteobacteria and Fusobacteria were identified as the dominant bacterial phyla in largemouth bass. These phyla are crucial for various physiological and biochemical processes, including growth and metabolism. The abundance of *Cetobacterium*, *Stenotrophomonas*, and *Pseudomonas* exhibited significant correlations with amino acids and fatty acids, highlighting their importance in promoting heterosis of hybrid largemouth bass. However, the mechanisms by which the aforementioned microbial taxa influence heterosis require further in-depth investigation.

## Figures and Tables

**Table 1:** Muscle proximate composition of four populations of largemouth bass.

Items	NC	NN	CN	CC
Moisture (g/100 g)	77.15 ± 0.42	76.73 ± 0.19	77.2 ± 0.43	77.38 ± 0.23
Ash (g/100 g)	1.23 ± 0.05 ^a^	1.2 ± 0.04 ^ab^	1.1 ± 0.00 ^b^	1.15 ± 0.03 ^ab^
Lipid (g/100 g)	1.35 ± 0.09 ^a^	0.88 ± 0.09 ^b^	1.2 ± 0.15 ^ab^	0.93 ± 0.10 ^b^
Protein (g/100 g)	19.85 ± 0.33	20.63 ± 0.17	19.93 ± 0.23	20.1 ± 0.30

Notes: NC and CN, hybrid groups; NN and CC, purebred groups. In each row, different lowercase letters indicate statistically significant difference.

**Table 2:** Muscle amino acid structure of various largemouth bass populations.

Amino Acid (g/100 g)	NC	NN	CN	CC
Aspartate (Asp)	1.73 ± 0.02 ^a^	1.74 ± 0.01 ^a^	1.66 ± 0.02 ^b^	1.57 ± 0.01 ^c^
* Threonine (Thr)	0.77 ± 0.02 ^a^	0.77 ± 0.01 ^a^	0.74 ± 0.01 ^b^	0.70 ± 0.00 ^c^
Serine (Ser)	0.62 ± 0.01 ^a^	0.61 ± 0.01 ^a^	0.60 ± 0.01 ^a^	0.56 ± 0.00 ^b^
Glutamate (Glu)	2.46 ± 0.05 ^a^	2.45 ± 0.03 ^a^	2.34 ± 0.04 ^a^	2.20 ± 0.02 ^b^
* Isoleucine (IIe)	0.80 ± 0.01 ^b^	0.84 ± 0.00 ^a^	0.77 ± 0.01 ^c^	0.74 ± 0.01 ^c^
* Leucine (Leu)	1.41 ± 0.02 ^a^	1.42 ± 0.01 ^a^	1.35 ± 0.02 ^b^	1.27 ± 0.00 ^c^
Tyrosine (Tyr)	0.57 ± 0.01 ^a^	0.57 ± 0.01 ^a^	0.54 ± 0.01 ^a^	0.50 ± 0.00 ^b^
* Phenylalanine (Phe)	0.74 ± 0.01 ^a^	0.75 ± 0.01 ^a^	0.71 ± 0.01 ^b^	0.68 ± 0.00 ^c^
* Lysine (Lys)	1.65 ± 0.03 ^a^	1.66 ± 0.01 ^a^	1.57 ± 0.02 ^b^	1.49 ± 0.00 ^c^
* Histidine (His)	0.39 ± 0.01 ^ab^	0.40 ± 0.01 ^a^	0.37 ± 0.01 ^bc^	0.35 ± 0.01 ^c^
Arginine (Arg)	1.04 ± 0.02 ^a^	1.05 ± 0.02 ^a^	1.01 ± 0.01 ^a^	0.94 ± 0.01 ^b^
Proline (Pro)	0.57 ± 0.00 ^a^	0.58 ± 0.01 ^a^	0.56 ± 0.01 ^ab^	0.54 ± 0.01 ^b^
Glycine (Gly)	0.85 ± 0.01	0.84 ± 0.02	0.83 ± 0.00	0.81 ± 0.02
Alanine (Ala)	1.07 ± 0.01 ^a^	1.07 ± 0.01 ^a^	1.02 ± 0.01 ^b^	0.98 ± 0.01 ^c^
Cystine (Cys)	0.22 ± 0.01 ^a^	0.20 ± 0.01 ^ab^	0.20 ± 0.01 ^ab^	0.19 ± 0.01 ^b^
* Valine (Val)	0.87 ± 0.01 ^b^	0.91 ± 0.00 ^a^	0.84 ± 0.01 ^c^	0.80 ± 0.00 ^d^
* Methionine (Met)	0.48 ± 0.01 ^a^	0.47 ± 0.02 ^a^	0.46 ± 0.02 ^a^	0.40 ± 0.00 ^b^
Total essential amino acid (EAA)	7.11 ± 0.07 ^a^	7.22 ± 0.06 ^a^	6.79 ± 0.09 ^b^	6.44 ± 0.03 ^c^
Total non-essential amino acids (NEAA)	9.12 ± 0.12 ^a^	9.12 ± 0.12 ^a^	8.75 ± 0.09 ^b^	8.30 ± 0.08 ^c^
Delicious amino acid (DAA)	6.11 ± 0.08 ^a^	6.10 ± 0.07 ^a^	5.85 ± 0.07 ^b^	5.57 ± 0.05 ^c^
Total amino acids (TAA)	16.23 ± 0.2 ^a^	16.33 ± 0.18 ^a^	15.53 ± 0.18 ^b^	14.73 ± 0.09 ^c^
Essential amino acids/total amino acids (EAA/TAA)	0.44 ± 0.00	0.44 ± 0.00	0.44 ± 0.00	0.44 ± 0.00

Note: Within a row, different letters indicate statistically significant difference; * indicates essential amino acids. EAA: essential amino acids; NEAA: non-essential amino acids; DAA: delicious amino acids (Asp, Glu, Gly, and Ala); TAA: total amino acids. NC and CN, hybrid groups; NN and CC, purebred groups.

**Table 3:** Muscle fatty acid structure of various largemouth bass populations (%).

Fatty Acid (%)	NC	NN	CN	CC
C14:0	0.83 ± 0.05 ^b^	1.04 ± 0.02 ^a^	1.19 ± 0.04 ^a^	1.21 ± 0.08 ^a^
C16:0	20.17 ± 0.88 ^a^	17.24 ± 0.24 ^b^	18.92 ± 0.50 ^ab^	17.91 ± 0.33 ^b^
C18:0	10.08 ± 0.45 ^a^	7.28 ± 0.40 ^b^	6.25 ± 0.28 ^b^	6.33 ± 0.62 ^b^
SFA	31.09 ± 1.25 ^a^	25.56 ± 0.16 ^b^	26.37 ± 0.68 ^b^	25.45 ± 0.60 ^b^
C16:1	1.89 ± 0.05 ^b^	2.11 ± 0.08 ^b^	2.87 ± 0.11 ^a^	2.72 ± 0.03 ^a^
C18:1n9c	19.73 ± 0.21 ^c^	20.79 ± 0.84 ^bc^	25.12 ± 0.77 ^a^	22.18 ± 0.17 ^b^
C20:1	0.82 ± 0.00 ^b^	0.80 ± 0.01 ^b^	0.94 ± 0.04 ^a^	0.90 ± 0.02 ^a^
C22:1n9	2.18 ± 0.10 ^ab^	2.26 ± 0.17 ^a^	1.81 ± 0.08 ^b^	1.94 ± 0.09 ^ab^
C24:1	1.82 ± 0.04 ^a^	1.17 ± 0.13 ^b^	1.15 ± 0.18 ^b^	0.88 ± 0.08 ^b^
MUFA	26.45 ± 0.32 ^c^	27.13 ± 0.63 ^bc^	31.88 ± 0.87 ^a^	28.62 ± 0.17 ^b^
C18:2n6c	19.08 ± 0.37 ^b^	22.91 ± 0.64 ^a^	22.19 ± 0.73 ^a^	23.40 ± 0.42 ^a^
C18:3n3	0.81 ± 0.03 ^b^	1.43 ± 0.03 ^a^	1.41 ± 0.06 ^a^	1.44 ± 0.07 ^a^
C20:2	0.91 ± 0.03 ^a^	0.79 ± 0.04 ^ab^	0.75 ± 0.06 ^b^	0.79 ± 0.04 ^ab^
C20:3n6	0.00 ± 0.00 ^b^	0.00 ± 0.00 ^b^	0.00 ± 0.00 ^b^	0.35 ± 0.03 ^a^
C20:4n6	1.88 ± 0.05 ^a^	0.78 ± 0.05 ^b^	0.96 ± 0.2 ^b^	1.07 ± 0.19 ^b^
C20:5n3 (EPA)	1.33 ± 0.02 ^b^	1.92 ± 0.06 ^a^	1.28 ± 0.04 ^b^	1.35 ± 0.09 ^b^
C22:6n3 (DHA)	18.46 ± 0.56 ^a^	19.48 ± 0.89 ^a^	15.16 ± 0.72 ^b^	17.54 ± 0.27 ^a^
PUFA	42.47 ± 0.93 ^b^	47.31 ± 0.6 ^a^	41.75 ± 0.87 ^b^	45.93 ± 0.51 ^a^
PUFA n-3	20.59 ± 0.60 ^b^	22.83 ± 0.91 ^a^	17.86 ± 0.69 ^c^	20.33 ± 0.35 ^b^
PUFA n-6	20.96 ± 0.36 ^c^	23.69 ± 0.62 ^b^	23.14 ± 0.54 ^b^	24.82 ± 0.24 ^a^
∑n-6/∑n-3	1.02 ± 0.02 ^b^	1.04 ± 0.06 ^b^	1.30 ± 0.06 ^a^	1.22 ± 0.02 ^a^

Note: In each row, different small letters indicate a statistically significant difference. DHA: docosahexaenoic acid; EPA: eicosapentaenoic acid; SFA: saturated fatty acids; MUFA: monounsaturated fatty acids; PUFA: polyunsaturated fatty acids; PUFA n-3: omega 3 polyunsaturated fatty acids; PUFA n-6: omega 6 polyunsaturated fatty acids. NC and CN, hybrid groups; NN and CC, purebred groups.

## Data Availability

The original contributions presented in this study are included in the article/supplementary material. Further inquiries can be directed to the corresponding author.

## Supplementary Materials

The following supporting information can be downloaded at: https://www.mdpi.com/article/10.3390/microorganisms13071449/s1.
